# Clinical Factors and Outcomes of Atypical Meningioma: A Population-Based Study

**DOI:** 10.3389/fonc.2021.676683

**Published:** 2021-05-26

**Authors:** Gui-Jun Zhang, Xiao-Yin Liu, Chao You

**Affiliations:** Department of Neurosurgery, West China Hospital, Sichuan University, Chengdu, China

**Keywords:** atypical meningioma, prognostic factor, treatment, SEER database, nomogram

## Abstract

**Objective:**

Atypical meningioma is a non-benign tumor, and its prognostic factors and treatment strategies are unclear.

**Methods:**

Patients with atypical meningioma, between 2004 and 2016, were collected from the Surveillance, Epidemiology, and End Results database. Then, we randomly divided patients into a training set and a validation set at a ratio of 8:2. The nomogram was constructed based on the multivariate Cox regression analyses. And the concordance index, calibration curves, and receiver operating character were used to assess the predictive ability of the nomogram. We divided the patient scores into three groups and constructed a survival curve using Kaplan–Meier analysis.

**Results:**

After our inclusion and exclusion criteria, 2358 patients were histologically diagnosed of atypical meningioma. The prognostic nomogram comprised factors of overall survival, including age, tumor size and surgery. The concordance index was 0.715 (95%CI=0.688-0.742) for overall survival in the training set and 0.688 (95%CI=0.629-0.747) for overall survival in the validation set. The calibration curves and receiver operating character also indicated the good predictability of the nomogram. Risk stratification revealed a statistically significant difference among the three groups of patients according to quartiles of risk score.

**Conclusion:**

Gross total resection is an independent factor for survival, and radiation after non-gross total resection potentially confers a survival advantage for patients with atypical meningioma.

## Introduction

Meningiomas constitute the most prevalent primary intracranial tumor, with an annual incidence of around 5 per 100,000 individuals, which account for approximately 30% of central nervous system, ranging from World Health Organization (WHO) grade I benign to WHO grade III malignant meningioma (1, 2). Atypical meningioma, as grade II, belongs to a distinctive category with a typical behavior, accounting for 8% of all meningiomas (3).

For treatment strategy in the atypical meningioma, an increasing number of reports advocated the critical importance of extensive resection as initial therapy (4, 5), which indicated the extent of resection affected prognosis. But some scholars suspected the role of aggressive resection on survival. On the other hand, limited literature existed over whether radiotherapy should be added into standard therapy regardless of the extent of resection for this non-benign tumor (6–9).

Given the controversy in the literature, and undetailed enunciation about prognostic factors for atypical meningioma from small series (10–12), we aimed to identify the prognostic factors to modify treatment strategies so as to improve the survival of patients with this tumor. Meanwhile, a nomogram model was established and validated for reliable estimation of 3-, 5-, and 8-year survival.

## Material and Methods

The National Cancer Institute’s Surveillance, Epidemiology and End Results (SEER) database includes incidence, patient demographics, clinic-pathological, treatment, and survival data from approximately 28% of the US cancer cases. This study cohort included adult patients more than 18 years old with histologically confirmed atypical meningioma. The extracted clinical information included in the following: patient ID, age at diagnosis, sex, race, year of diagnosis, primary site, laterality, tumor size, diagnostic confirmation, surgery at the primary site, radiation code, survival months, and vital status. The exclusion criteria were as follows (**Supplementary Figure 1** **)**: 1) missing critically clinical patient information; 2) not confirmed histologically atypical meningioma; 3) patient survival time equally to 0 month; 4) not primary sequence only; 5) patients without surgical resection.

After filtering the data, additional classification was performed: sex (female *vs*. male), location (cerebral menings *vs*. non-cerebral menings), laterality (left *vs*. right *vs*. bilateral *vs*. others), surgery (gross total resection (GTR) *vs*. non-GTR), and radiation (yes *vs*. no). The age and tumor size were divided into subgroups using the receiver operating character (ROC). For the extent of resection, “radical resection” was considered GTR; “biopsy”, “subtotal resection”, and “partial resection” were considered non-GTR. The endpoint was defined as overall survival (OS).

### Construction and Validation of the Nomogram

The enrolled patients were randomly divided into training and validation sets at a ratio of 8:2, and the clinical information of two groups were described. Survival analysis were performed using univariate and multivariate proportional hazard models. Univariate analysis was performed first and variables were inclusion for multivariate analysis if the univariate p value<0.15.

The nomogram was established to estimate 3-, 5-, and 8-year OS rates for patients with atypical meningioma, and then nomogram models were internally validated. To verify the discrimination ability of the nomogram, we used concordance index (C-index) and ROC in the training and validation sets. In addition, calibration curve was performed to assess the consistency between actual prognosis and predicted survival.

Categorical variables were compared with the Chi-square or Fisher’s exact test, and continue variables were compared with the independent-samples student’s t-test. In addition, we calculated the scores of each patient in the training cohorts based on the nomogram models. Then, we divided the training set into three groups according to the total score of each patient, and constructed the survival curve and log-rank test to compare the OS of patients in the different groups.

### Statistical Analysis

Statistical analysis was performed using R statistical software (version 3.6.3) and SPSS software (version 25.0; IBM Corp, Armonk, NY, USA), p<0.05 was considered statistically significant.

## Results

After our inclusion and exclusion criteria, 2358 patients with histologically-identified atypical meningioma in the SEER database and were included in our further analysis. The best cut-off value for the age and the tumor size were determined to be 67.5 years and 52.5 mm, respectively. The clinical variables of patients were in the training and validation sets (**Table 1**).

**Table 1:** Demographic characteristics of included 2358 cases with atypical meningioma.

Variable	Total	Training set	Validation set	P value
	n (%)	n (%)	n (%)	
	2358	1888 (80.1)	470 (19.9)	
Sex				0.239*
Male	992 (42.1)	783 (41.5)	209 (44.5)	
Female	1366 (57.9)	1105 (58.5)	261 (55.5)	
Race				0.554*
White	1738 (73.7)	1385 (73.4)	353 (75.1)	
Black	325 (13.8)	258 (13.7)	67 (14.3)	
Other	275 (11.7)	229 (12.1)	46 (9.8)	
Unknown	20 (0.8)	16 (0.8)	4 (0.9)	
Age, years				0.192^†^
Range	20-93	20-93	21-90	
Mean	59.2 ± 15.0	59.4 ± 15.1	58.4 ± 14.8	
Median	60	61	60	
Location				0.981*
Cerebral meninges	2165 (91.8)	1731 (91.6)	434 (92.3)	
Meninges, NOS	165 (7.0)	132 (7.0)	33 (7.0)	
Cerebrum	2 (0.1)	2 (0.1)	0	
Frontal lobe	13 (0.6)	11 (0.6)	2 (0.4)	
Temporal lobe	2 (0.1)	2 (0.1)	0	
Parietal lobe	2 (0.1)	2 (0.1)	0	
Occipital lobe	2 (0.1)	2 (0.1)	0	
Cerebellum	2 (0.1)	2 (0.1)	0	
Brain, NOS	2 (0.1)	1 (0.1)	1 (0.2)	
Overlapping lesion of brain	2 (0.1)	2 (0.1)	0	
Pineal gland	1 (0)	1 (0.1)	0	
Year of diagnosis				0.846*
2004-2010	974 (41.3)	778 (41.2)	196 (41.7)	
2011-2016	1384 (58.7)	1110 (58.8)	274 (58.3)	
Laterality				0.164*
Bilateral	51 (2.2)	36 (1.9)	15 (3.2)	
Left	1072 (45.5)	868 (46.0)	204 (43.4)	
Right	1032 (43.8)	820 (43.4)	212 (45.1)	
Not a paired site	97 (4.1)	78 (4.1)	19 (4.0)	
Only one side	3 (0.1)	1 (0.1)	2 (0.4)	
Paired site	103 (4.4)	85 (4.5)	18 (3.8)	
Tumor size, mm				0.078†
Range	5-145	6-145	5-120	
Mean	49.3 ± 17.2	49.6 ± 17.2	48.0 ± 16.9	
Median	49	49	47	
Surgery				0.540*
Non-GTR	994 (42.2)	790 (41.8)	204 (43.4)	
GTR	1364 (57.8)	1098 (58.2)	266 (56.6)	
Radiation				0.076*
Yes	660 (28.0)	513 (27.2)	147 (31.3)	
None	1698 (72.0)	1375 (72.8)	323 (68.7)	

*Chi-square test.

^†^Independent t-test.

### The Training Set

Of 1888 patients in the training set, there was 1105 (58.5%) female patients and 783 (41.5%) male patients, with a female-male ratio of 1.4. The median age at diagnosis was 61 years, with an age range of 20-93 years. The majority of patients (n=1110, 58.8%) were diagnosed after year 2011. The 3-, 5-, and 8-year OS for all patients by Kaplan-Meier analysis were 87.1%, 78.9%, and 67.7%, respectively. GTR was achieved in 1098 (58.2%) patients, and non-GTR was achieved in 790 (41.8%) patients. Most patients (n=1375, 72.8%) declined adjuvant radiotherapy.

**Figure 1:**
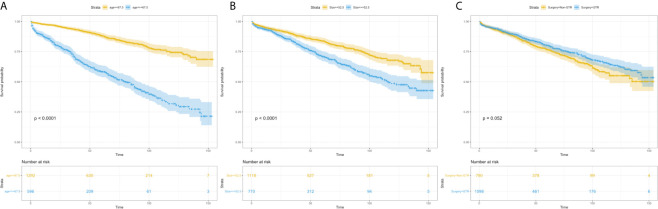
Kaplan–Meier estimated overall survival in patients with atypical meningioma that were: age<67.5 years *vs*. age≥67.5 years **(A)** tumor size<52.5mm *vs*. tumor size≥52.5mm **(B)** non-gross total resection *vs*. gross total resection **(C)**.

**Figure 2:**
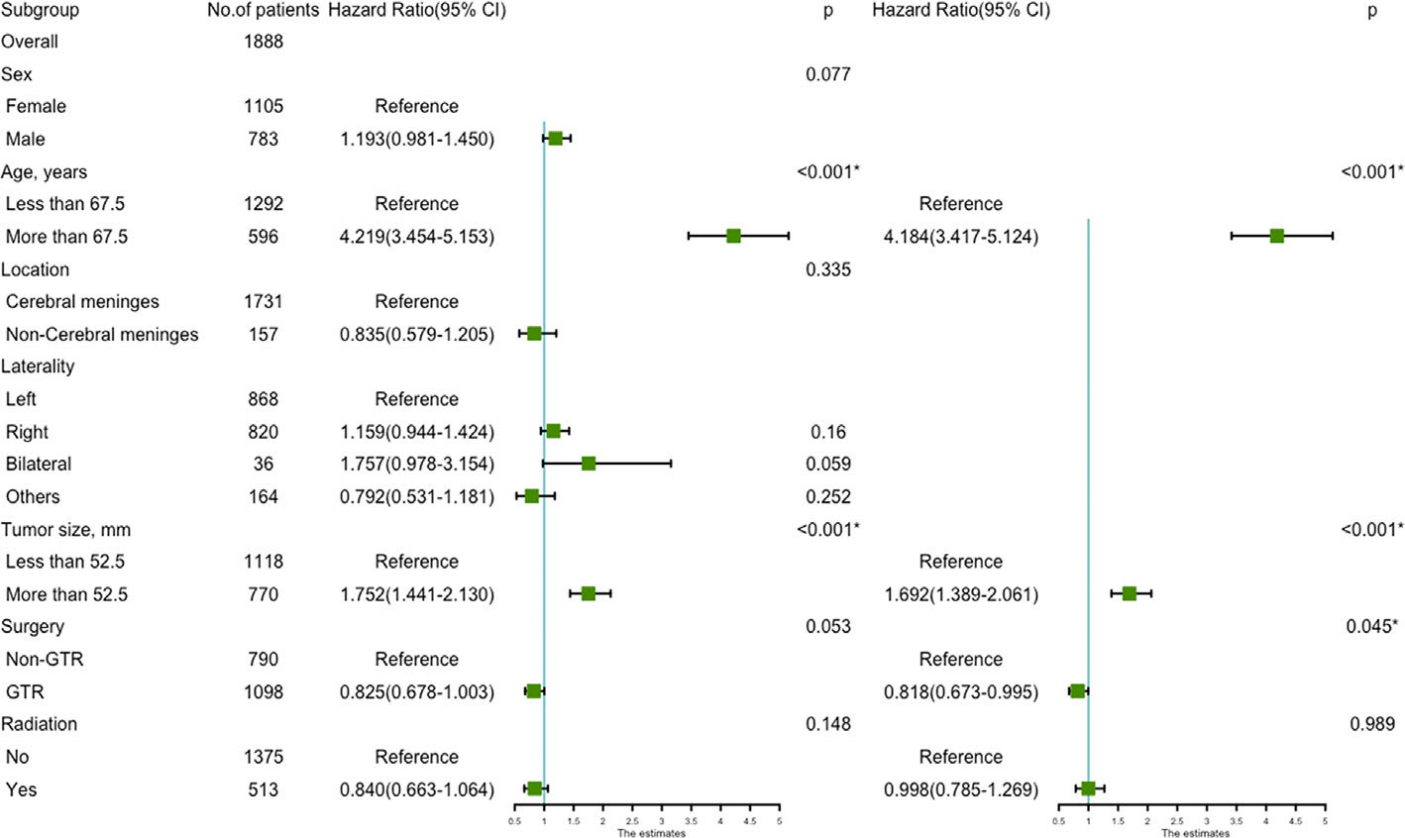
The forest map of Cox regression analysis. Univariate and multivariate Cox regression analyses and estimating the risk factors for overall survival in the training set. *Means *P* < 0.05.

Using univariate analysis, factors significantly predicting worse OS included age more than 67.5 years (HR=4.219, 95%CI=3.454-5.153; p<0.001) (**Figure 1A**), and tumor size more than 52.5mm (HR=1.752, 95% CI=1.441-2.130; p<0.001) (**Figure 1B**); factors trending toward a better OS included male (HR=1.193, 95%CI=0.981-1.450; p=0.077) and GTR (HR=0.825, 95%CI=0.678-1.003; p=0.053) (**Figure 1C**). By multivariate analysis, older age (HR=4.184, 95%CI=3.417-5.124; p<0.001), larger tumor size (HR=1.692, 95%CI=1.389-2.061; p<0.001) significantly predicted worse OS, and GTR (HR=0.818, 95% 220 CI=0.673-0.995; p=0.045) was an independent favorable factor of better OS (**Figure 2**).

**Figure 3:**
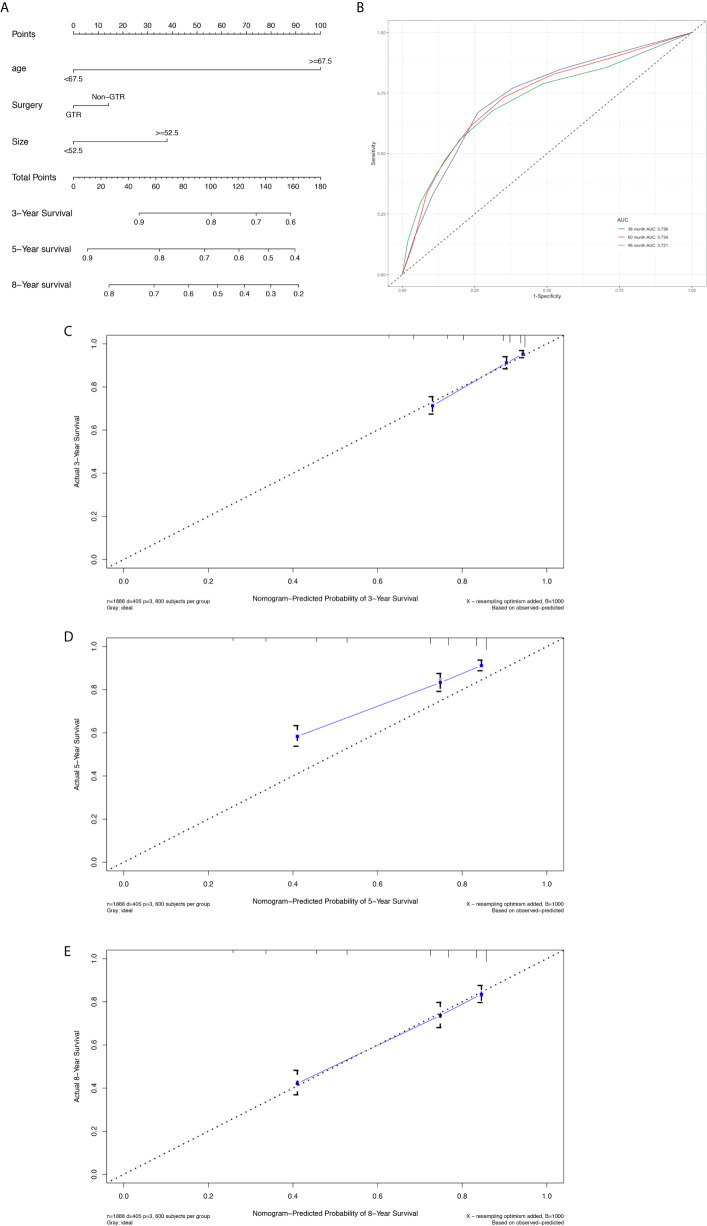
Nomogram used to predict the 3-, 5- and 8-year OS rates of patients with atypical meningioma **(A)**. AUC curve of receiver operating character of the nomogram for predicting the 3-, 5- and 8- year overall rates of patients with atypical meningioma from the training set **(B)**. Calibration curve of the nomogram for predicting the 3- **(C)**, 5- **(D)** and 8- **(E)** year OS rates of patients with atypical meningioma from the training set.

The prognostic nomogram (**Figure 3A**) comprised all significant factors of OS based on the multivariate analysis. The C-index for OS was 0.715 (95%CI=0.688-0.742). AUCs for ROC curves (**Figure 3B**) and the calibration plot (**Figures 3C–E**) for the probability of survival at 3, 5, and 8 years displayed an ideal agreement between the prediction and actual observations by nomogram, which suggested that the best discriminative ability of nomogram models.

### The Validation Set

A total of 470 patients were included in the validation set. The median age at diagnosis was 60 years (range, 21-90 years), and the sex distribution showed a slight female predominance (n=261, 55.5%). 75.1% of patients (n=353) were white, 14.3% of patients (n=67) were black, and 10.7% of patients (n=50) were others and unknown. The median tumor size was 47mm, ranging from 5 to 120mm. Therapeutically, 266 (56.6%) patients underwent GTR and 147 (31.3%) patients received adjuvant radiation. We did not get the median OS. The 3, 5-, and 8-year OS rates were 87.5%, 80.3% and 69.0%, respectively, ranging from 1 to 155 months.

**Figure 4:**
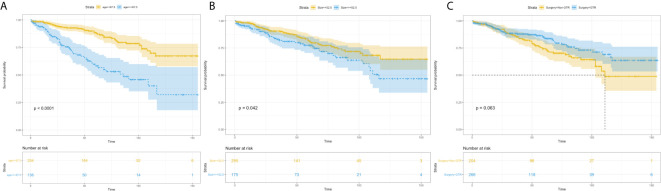
Kaplan–Meier estimated overall survival in patients with atypical meningioma that were: age<67.5 years *vs*. age≥67.5 years **(A)** tumor size<52.5mm *vs*. tumor size≥52.5mm **(B)** non-gross total resection *vs*. gross total resection **(C)**.

**Figure 5:**
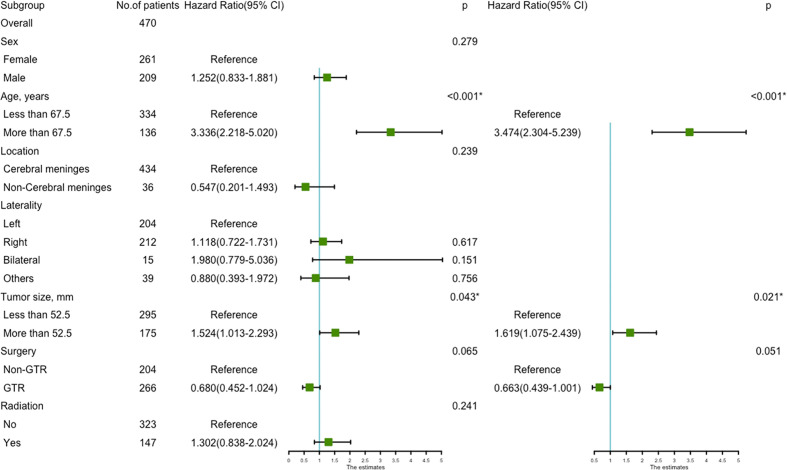
The forest map of Cox regression analysis. Univariate and multivariate Cox regression analyses and estimating the risk factors for overall survival in the validation set. *Means *P* < 0.05.

Univariate analysis revealed that age more than 67.5 years (HR=3.336, 95%CI=2.218-5.020; p<0.001) (**Figure 4A**), and tumor size more than 52.5 mm (HR=1.524, 95%CI=1.013-2.293; p=0.043) were significantly associated with worse survival (**Figure 4B**). GTR (HR=0.680, 95%CI=0.452-1.024; p=0.065) trending toward a better survival (**Figure 4C**). Multivariate analysis revealed that age more than 67.5 years (HR=3.474, 95%CI=2.304-5.239; p<0.001) and tumor size more than 52.5mm (HR=1.619, 95%CI=1.075-2.439; p=0.021) remained statistical significance (**Figure 5**).

**Figure 6:**
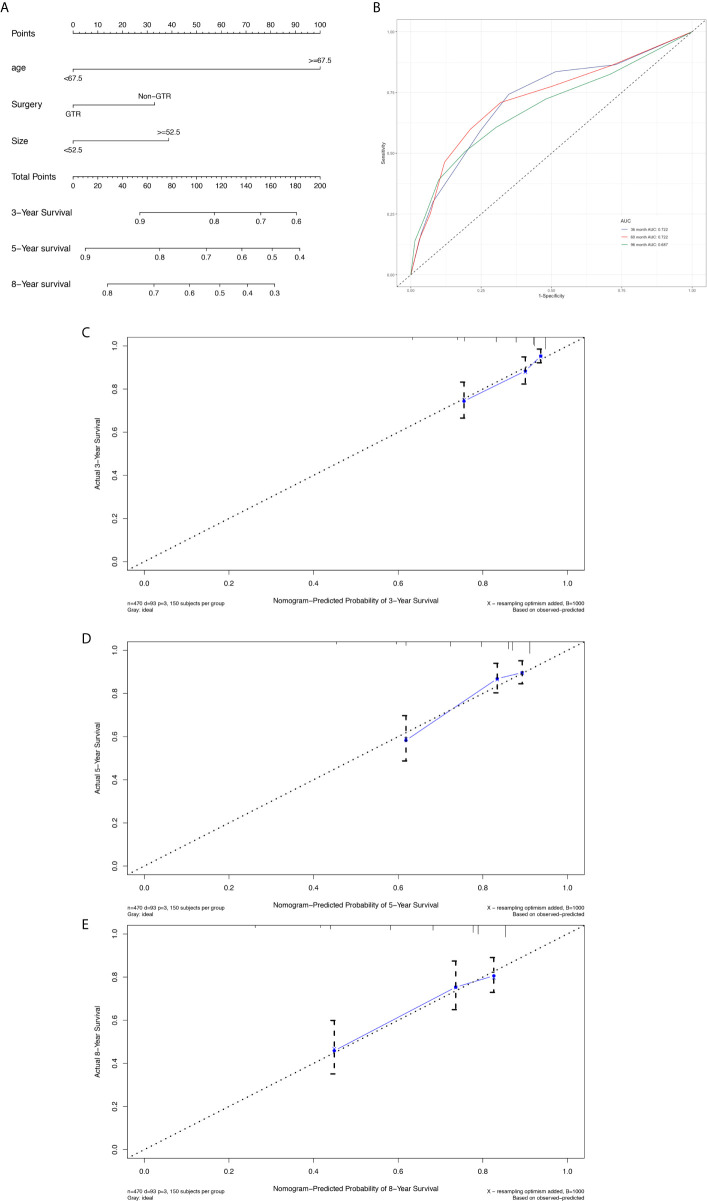
Nomogram used to predict the 3-, 5- and 8-year OS rates of patients with atypical meningioma **(A)**. AUC curve of receiver operating character of the nomogram for predicting the 3-, 5- and 8- year overall rates of patients with atypical meningioma from the validation set **(B)**. Calibration curve of the nomogram for predicting the 3- **(C)**, 5- **(D)** and 8- **(E)** year OS rates of patients with atypical meningioma from the validation set.

The prognostic nomogram (**Figure 6A**) comprised two significant factors (age and tumor size) and one therapeutic factor (surgery, p=0.051). The C-index was 0.688 (95%CI=0.629-0.747) for OS, the AUCs for ROC curves indicated that nomogram models had best risk discriminative ability (**Figure 6B**), and the predicted calibration curves were closed to the standard curves for 3-, 5-, and 8-year survival for OS (**Figures 6C–E**).

### Risk Stratification

The total score was calculated for each patient in the training and validation sets, and the scores were divided into three subgroups for OS (0-14.1, 14.1-52, 52-152, training set; 0, 33-71.7, 71.7-171.7, validation set) to display different outcomes (**Supplementary Figure 2A** and **Figure 2B**).

To explore the role of adjuvant radiotherapy in addition to STR in survival, the entire cohort was divided into GTR and non-GTR groups. In the both GTR and non-GTR groups, there was no significant difference in survival between surgery alone and surgery with radiation. (Chi-square=0.018, p=0.893, log rank; GTR group). But, in the non-GTR group, surgery with radiation toward reaching a significantly increased survival compared with surgery alone (111.7 *vs*. 107.2 months) (Chi-square=2.363, p=0.124, log-rank; non-GTR group).

## Discussion

Prior to this study, to our best knowledge, there was no nomogram model for atypical meningioma, and because of this, a nomogram was developed based on a SEER database in this study. We identified the age, tumor size and surgical treatment as significant prognostic factors of OS. The nomogram established in this study not only was of high predictive power but also significant for clinical treatment.

Female sex showed a predominance in terms of incidence, and it conferred a toward OS advantage in univariate analysis. We were aware that most benign meningioma had slight prediction to affect female patients. But the potential relation between sex and survival in atypical meningioma need to be further explored. Older patients more than 67.5 years had an increased risk of mortality, in our study, compared with those who were under 67.5 years, which was consistent with previous studies (13–15).

A larger tumor size was found to be a paramount consideration associated with worse survival, which has been rarely reported by others. Larger tumor size had greater mass effect and more non-tumor tissues were involved at a high risk, which caused a difficulty for total resection.

Zaher et al. (13) reported on 44 patients with atypical meningioma who were surgically treated between 2009 and 2012, GTR was achieved in 16 cases and subtotal resection (STR) was achieved in 22 cases. A significant difference in OS between GTR and STR was observed in their study. Also, in a large series of 302 cases with atypical meningioma (16), the 3- and 5- year OS rates for GTR were 94.0% and 83.9%, respectively, and the 3- and 5- year OS rates for STR were 84.5% and 70.0%, respectively. Our study confirmed the findings of previous literature regarding complete resection statistically associated with increase in survival, which indicated that extent of resection was the most significant factor for outcome. On the contrary to us, some studies did not support the role of GTR in OS, although they demonstrated that GTR as a favorable factor of better progression-free survival (PFS) (17). Moreover, many studies assessed the effect of surgery on PFS following surgery, rather than OS after surgery (7, 18). Our studies made up for the lack of association between surgery and OS in the historical process.

The effect of adjuvant radiotherapy on survival of patients with atypical meningioma remained controversial (14). In our study, adjuvant radiation was not statistically related to increased OS. A multi-institutional study of 166 patients with grade II meningioma (atypical meningioma, n=149) by Purand et al. (19) over 14 years showed radiation did not affect OS. In another series of 99 cases with atypical meningioma, 72.7% patients received adjuvant radiotherapy. A significant difference, between surgery alone and surgery with radiotherapy, was not observed in either PFS or OS (20). In addition, there was an observe trend toward reduced risk of mortality with non-GTR+ radiotherapy compared with non-GTR alone, but it did not reach a significance. Despite a large sample size, we failed to prove the significant role of radiation in addition to non-GTR in survival. While some studies showed benefits to adjuvant radiation only in patients who underwent STR, rather than GTR (21–23). But in MK et al. and HP et al. studies, they found that radiation after even GTR could contribute to an improvement in PFS (24, 25).

There were several limitations of this study. The nomogram was established based on SEER database in the United States, which was probably not representation of patients worldwide with atypical meningioma. Moreover, considering a retrospective study, some critical prognostic factors were not included in our study due to the limitations of SEER database, such as the clinical presentation, bone/brain invasion and tumor markers, which might affect survival. Additionally, it was unable to stratify for Simpson grades and tumor location (convexity *vs*. non-convexity) that might be potential factors of survival.

## Conclusion

The proposal nomogram in this study accurately predicted the OS. Accordingly, GTR is an independent factor for survival, and radiation after non-GTR potentially confers a survival advantage for patients with atypical meningioma.

## Data Availability Statement 

The original contributions presented in the study are included in the article/**Supplementary Material**. Further inquiries can be directed to the corresponding author.

## Author Contributions

Conception and experimental design: all authors. Acquisition of data: G-JZ and X-YL. Analysis and interpretation of data: all authors. Drafting the article: G-JZ. Critically revising the article: all authors. Study supervision: all authors. All authors contributed to the article and approved the submitted version.

## Funding

Supported by 1.3.5 project for disciplines of excellence, West China Hospital, Sichuan University (NO. 2018HXFH010).

## Conflict of Interest

The authors declare that the research was conducted in the absence of any commercial or financial relationships that could be construed as a potential conflict of interest.

## Supplementary Material

Supplementary Figure 1 Study flow diagram

Supplementary Figure 2 Kaplan–Meier curves of risk group stratifications within the training set for OS **(A)** and validation set for OS **(B)**.

## Abbreviations

C-index, concordance index; GTR, gross total resection; OS, overall survival; PFS, progression-free survival; ROC, receiver operating characteristic; SEER, Surveillance, Epidemiology and End Results; STR, subtotal resection; WHO, World Health Organization.
