# Supplementary figures and images for: Clinical Factors and Outcomes of Atypical Meningioma: A Population-Based Study
Source: Front Oncol. 2021 May 26;11:676683. doi: 10.3389/fonc.2021.676683 (PMC8187879; doi:10.3389/fonc.2021.676683)

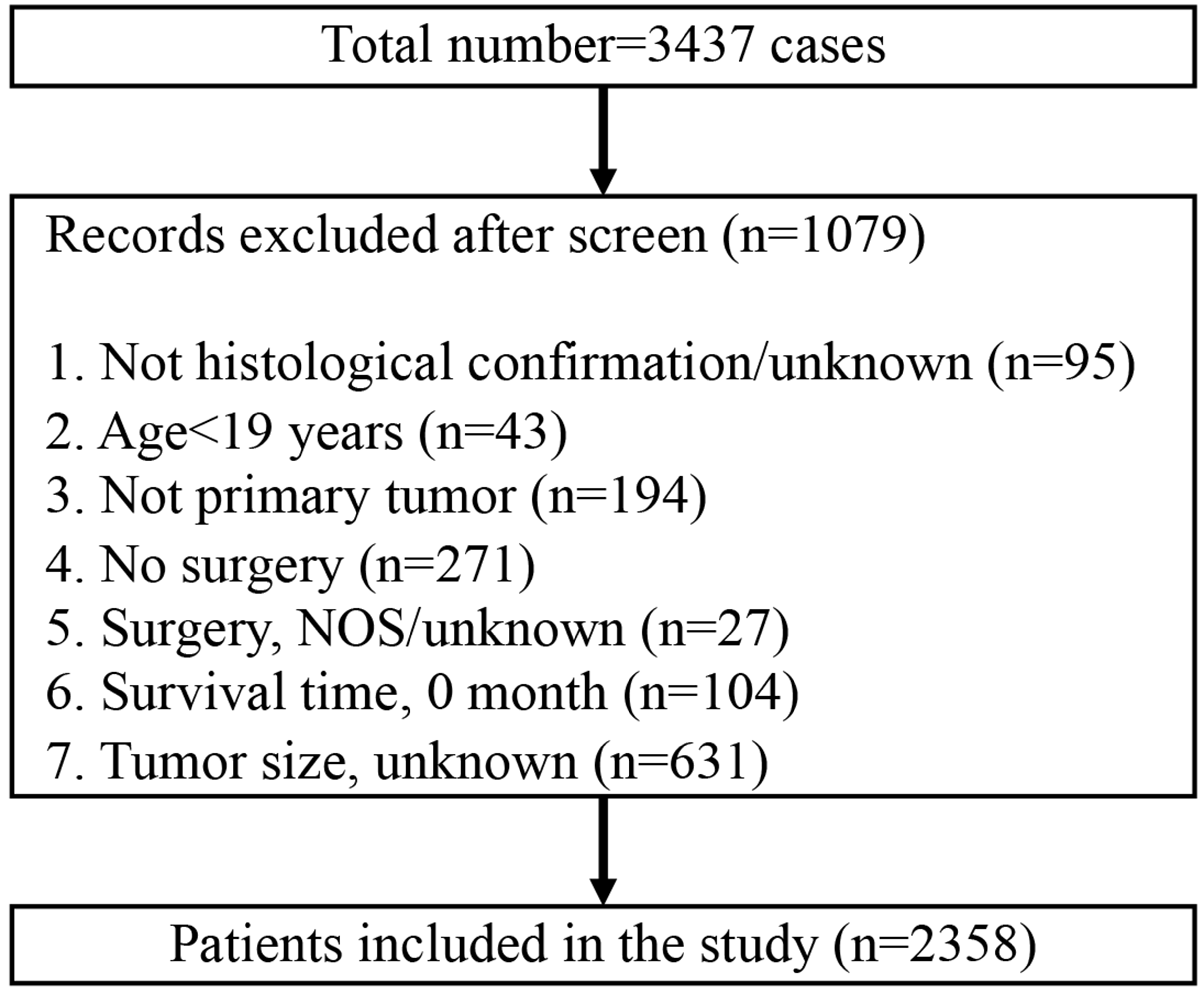

Supplement: Supplementary Figure 1 — Study flow diagram [file Image_1.tif]
